# Proteomic analysis of the inhibitory effect of epigallocatechin gallate on lipid accumulation in human HepG2 cells

**DOI:** 10.1186/1477-5956-11-32

**Published:** 2013-07-18

**Authors:** Zhonghua Liu, Qin Li, Jianan Huang, Qionglin Liang, Yujun Yan, Haiyan Lin, Wenjun Xiao, Yong Lin, Sheng Zhang, Bin Tan, Guoan Luo

**Affiliations:** 1 Department of Chemistry, Tsinghua University and Key Laboratory of Biological Organic Phosphorus and Chemical Biology, Ministry of Education, Beijing 100084, China; 2 Hunan Provincial Key Laboratory of Crop Germplasm Innovation and Utilization and Key Laboratory of Tea Science, Ministry of Education, Hunan Agricultural University, Changsha, Hunan 410128, China; 3 National Research Center of Engineering & Technology for Utilization of Botanical Functional Ingredients, Changsha, Hunan 410128, China

**Keywords:** Proteomics, (−)-Epigallocatechin-3-gallate, HepG2 cells, Lipid accumulation

## Abstract

**Background:**

(−)-Epigallocatechin-3-gallate (EGCG), the most abundant catechin found in green tea, effectively reduces body weight and tissue and blood lipid accumulation. To explore the mechanism by which EGCG inhibits cellular lipid accumulation in free fatty acid (FFA) induced HepG2 cell culture, we investigated the proteome change of FFA-induced HepG2 cells exposed to EGCG using two-dimensional gel electrophoresis and mass spectrometry.

**Results:**

In this study, 36 protein spots showed a significant change in intensity by more than 1.5-fold from the control group to the FFA group and from the FFA group to the FFA + EGCG group. Among them, 24 spots were excised from gels and identified by LC-MS/MS. In total, 18 proteins were successfully identified. All identified proteins were involved in lipid metabolism, glycometabolism, antioxidant defense, respiration, cytoskeleton organization, signal transduction, DNA repair, mRNA processing, iron storage, or were chaperone proteins. This indicated that these physiological processes may play roles in the mechanism of inhibition of lipid accumulation by EGCG in FFA-induced HepG2 cells. Western blotting analysis was used to verify the expression levels of differentially expressed proteins, which agree with the proteomic results.

**Conclusions:**

From the proteomic analysis, we hypothesized that EGCG reduced cellular lipid accumulation in FFA-induced HepG2 cells through the activation of AMP-activated protein kinase (AMPK) resulting from the generation of reactive oxygen species (ROS). The induction of ROS may be a result of EGCG regulation of the antioxidant defense system. Activation of AMPK shifted some FFA toward oxidation, away from lipid and triglyceride storage, and suppressed hepatic gluconeogenesis. The findings of this study improve our understanding of the molecular mechanisms of inhibition of lipid accumulation by EGCG in HepG2 cells.

## Background

Nonalcoholic fatty liver disease (NAFLD), defined by excessive liver fat accumulation related to metabolic syndrome, is a leading cause of progressive liver disease. NAFLD is a clinicopathological term that encompasses a disease spectrum ranging from simple lipid accumulation in hepatocytes (hepatic steatosis) to hepatic steatosis with inflammation (steatohepatitis), fibrosis, and cirrhosis [1]. Excess hepatic lipid accumulation is associated with nutritional factors, drugs, and multiple genetic defects in energy metabolism.

Green tea is widely consumed throughout the world, especially in East Asian countries. Research indicates that green tea is beneficial to health and many components of tea have specific health-promoting effects [2,3]. Studies have suggested that (−)-epigallocatechin-3-gallate (EGCG), the most abundant catechin found in green tea, could effectively reduce body weight and tissue and blood fat accumulation [4,5]. In high-fat-fed mice, EGCG decreased liver weight, liver triglycerides, plasma alanine aminotransferase concentrations, lipid accumulation in hepatocytes [6], and reduced the development of experimental nonalcoholic steatohepatitis through its effect on lipid metabolism [7]. EGCG treatment could effectively reduce fatty liver incidence, liver damage, and liver triglyceride levels in Male C57BL/6J mice fed with a high-fat, Western-style diet [8]. These beneficial effects of EGCG are associated with decreased lipid absorption and reduced levels of inflammatory cytokines. Previous studies have suggested that EGCG and green tea might modulate expression of lipid metabolism-related genes. EGCG treatment up-regulated several genes related to fat oxidation and thermogenesis, including liver acyl-CoA oxidase (AOX), medium-chain acyl-CoA dehydrogenase (MCAD), muscle uncoupling protein-2 (UCP2) and uncoupling protein-3 (UCP3), and fatty acid translocase [9-11]. Moreover, EGCG treatment down-regulated several genes related to fatty acid synthesis and storage in the liver and white adipose tissue, including acetyl-CoA carboxylase (ACC), fatty acid synthase (FAS), malic enzyme (ME), glucose-6-phosphate dehydrogenase (G6PDH), glycerol-3-phosphate dehydrogenase (G3PDH), and stearoyl-CoA desaturase-1 (SCD1) [12-15].

Despite the amount of work on EGCG, there is little proteomic information available on EGCG inhibiting lipid accumulation in free fatty acid (FFA) induced HepG2 cells. To investigate the inhibitory effect of EGCG on FFA-induced lipid accumulation, HepG2 cells were exposed to FFA co-treated with 50 μM EGCG. The proteome changes of FFA-induced HepG2 cells were investigated by two-dimensional gel electrophoresis (2-DE) combined with matrix-assisted laser desorption ionization time-of-flight mass spectrometry (MALDI-TOF/TOF MS). The objective of this study was to obtain an improved understanding of the mechanism by which EGCG inhibits lipid accumulation in FFA-induced HepG2 cells.

## Results

### Effect of EGCG on FFA-induced intracellular lipid accumulation in HepG2 cells

**Figure 1:**
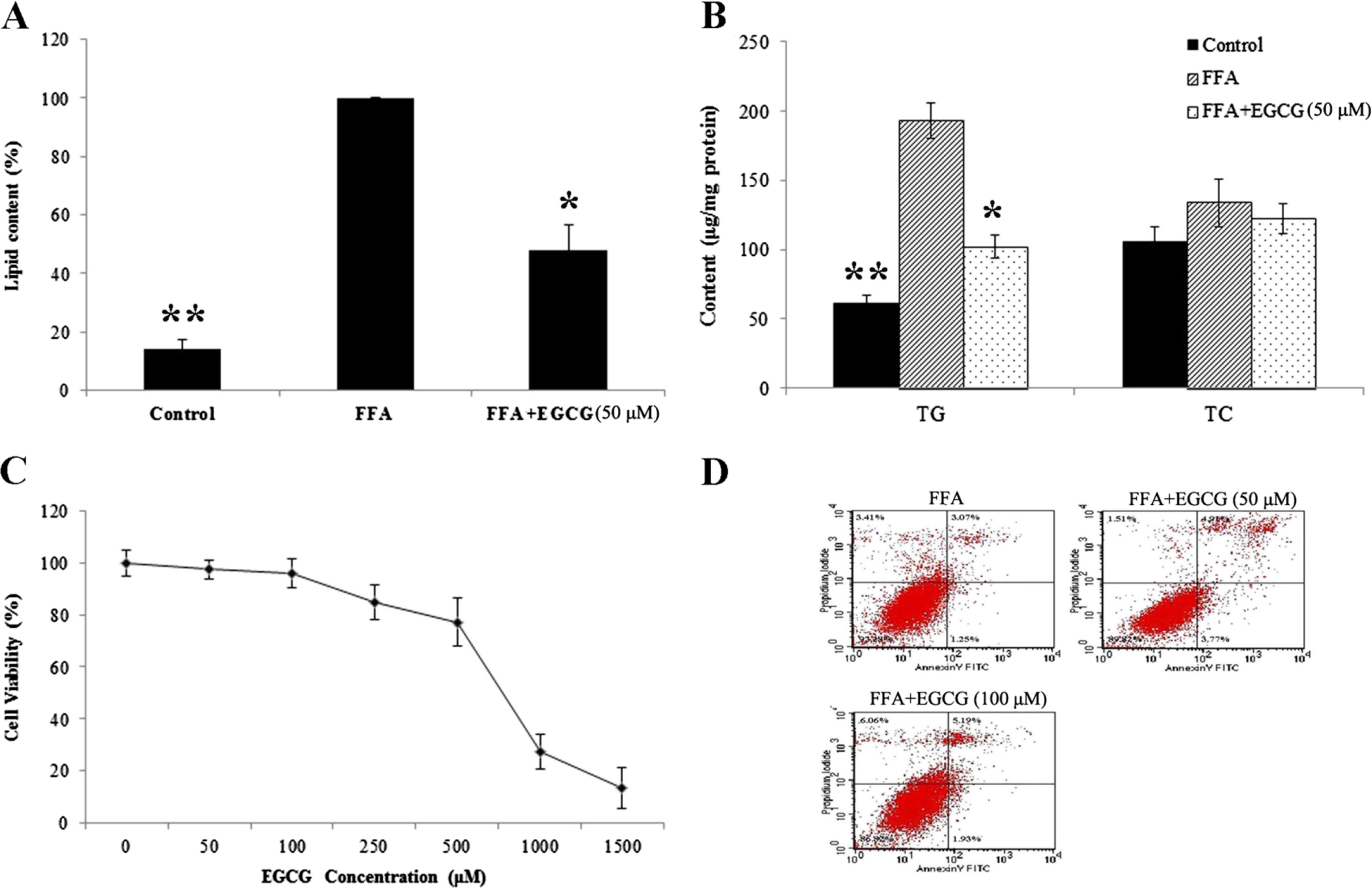
**EGCG reduced FFA-induced intracellular lipid accumulation in HepG2 cells.** Lipid content detected by a quantitative Oil Red O dye method **(A)**, Triglyceride and cholesterol levels in FFA-overloaded HepG2 cells in different treatment groups **(B)**, The effect of EGCG on FFA-overloaded HepG2 cells. Proliferation and apoptosis were assessed by MTT assay **(C)** and PI/annexin V staining **(D)**.

Fatty liver results from an imbalance between lipid availability and lipid metabolism. Palmitic and oleic acids are the most abundant FFA in liver triglycerides in both normal subjects and patients with NAFLD [16]. Exposure of HepG2 cells to exogenous FFA leads to significant intracellular lipid accumulation [17]. HepG2 cells loaded with 1 mM FFA mixture (oleic acid [OA] and palmitic acid [PA], 2:1) mimics benign chronic steatosis in humans [18]. As shown in Figure 1A, the intracellular lipid content could be significantly lowered by treatment with 50 μM EGCG. This suggested that EGCG could significantly inhibit FFA-induced intracellular lipid accumulation in HepG2 cells. This result was also confirmed by the quantification of intracellular triglycerides and cholesterol contents. In Figure 1B, EGCG treatment showed significantly lower triglyceride levels (*p* ≤ 0.05). The content of cholesterol was also lowered but not significantly. The effects of EGCG on FFA-stimulated HepG2 cell viability were examined by MTT assay and annexin V staining. The results showed that cell viability was not compromised by 50 μM EGCG treatment after 24 h of exposure (Figure 1C and D).

### Change in protein abundance in the three groups

**Figure 2:**
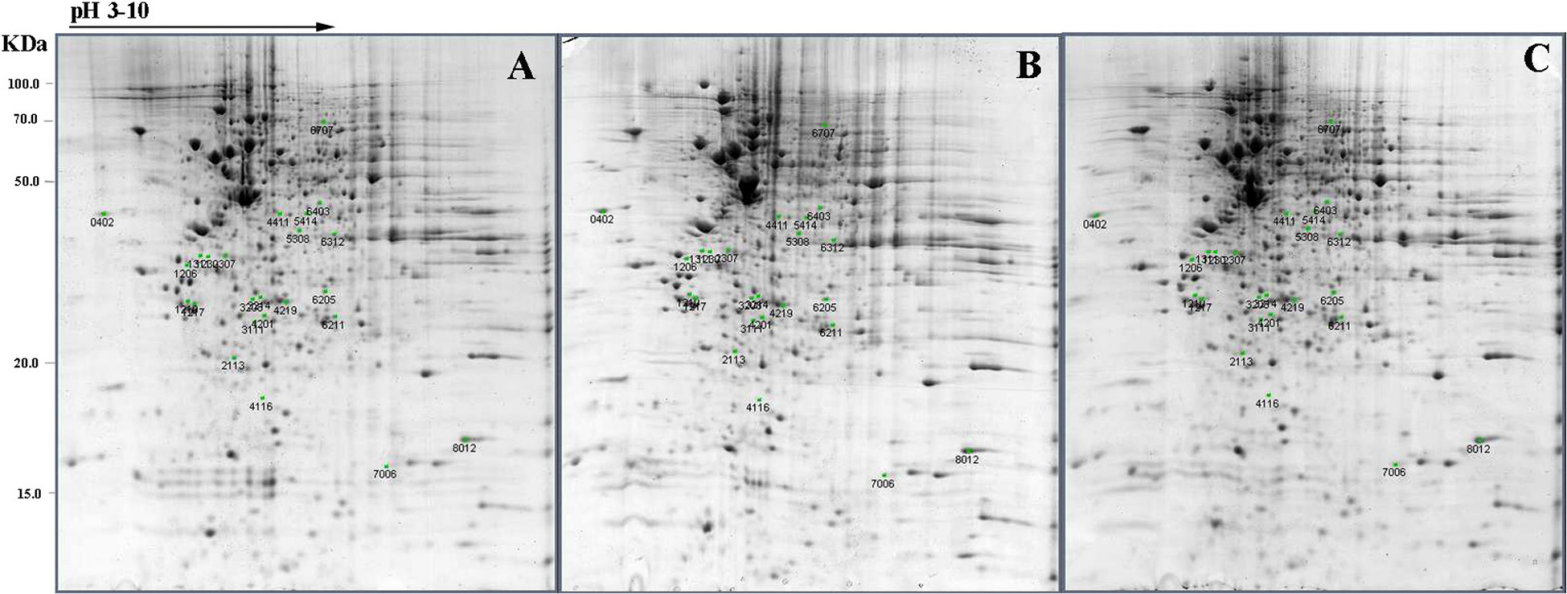
**Comparison of 2-D electrophoresis analysis of the proteins expressed in FFA-stimulated HepG2 cells derived from different treatment groups.** Control **(A)**, FFA **(B)**, FFA + EGCG **(C)**. Proteins that exhibited a significant expression change (≥ 1.5-fold, *p* ≤ 0.05) from the control group to FFA group while from the FFA group to FFA + EGCG group are labeled in the figures.

To explore the inhibitory effect of EGCG on FFA-induced lipid accumulation in HepG2 cells, the proteomes of the control, FFA-induced (FFA) and FFA-induced co-treated with 50 μM EGCG groups (FFA + EGCG) were analyzed by 2-DE (Figure 2). Among the tested samples, more than 800 protein spots were reproducibly detected with PDQuest 8.0.1 software on Coomassie Brilliant Blue (CBB) G-250-stained gels. The control and FFA + EGCG groups had 142 and 151 protein spots that showed a significant change in expression level in when compared with the FFA group, respectively. Moreover, 36 protein spots showed a significant change in intensity by more than 1.5-fold from the control group to the FFA group and from the FFA group to the FFA + EGCG group. Among them, 24 spots that changed at least 2.0-fold were excised from gels and identified by MALDI-TOF/TOF MS. In total, 18 protein spots were successfully identified and the results are summarized in Table 1. The functions of the differentially expressed proteins were obtained using their protein accession numbers from the SwissProt/NCBI protein function summary. All identified proteins in this study were involved in multiple functional groups (Table 1), including lipid metabolism, glycometabolism, antioxidant defense, respiration, cytoskeleton organization, signal transduction, DNA repair, mRNA processing, and iron storage, or were chaperone proteins.

**Table 1:** Differentially expressed proteins identified by MS or MS/MS

**Spot no.**	**Protein name**	**Accession no.**^**a**^	**Theoretical mr/pI**	**MOWSE score**	**Sequence coverage (%)**	**Protein expression**^**b**^			**Biological function**
						**A**	**B**	**C**	
**402**	Keratin 1	H6VRF8	66184/8.15	118	13				Cytoskeleton
**1210**	Lysosomal protective protein	P10619	34065/5.49	211	11				Glycometabolism
**1217**	14-3-3 protein gamma	P61981	28325/4.80	350	30				Signal transduction
**1311**	Ubiquitin thioesterase OTUB1	Q96FW1	26652/5.22	331	31				DNA repair
**2307**	Serine/arginine-rich splicing factor 7	Q16629	26224/11.77	161	15				mRNA processing
**3111**	Probable G-protein coupled receptor 179	Q6PRD1	260635/5.54	63	6				Signal transduction
**3208**	Prohibitin	Q6PUJ7	29859/5.57	154	23				Chaperone proteins
**3214**	C447E6.1 (Nucleotide binding protein 1)	Q2YS46	28604/5.89	65	16				Chaperone proteins
**4116**	Ferritin light chain	P02792	18818/5.65	158	36				Iron storage
**4201**	Phosphoserine phosphatase	P78330	25163/5.53	110	22				Protein phosphorylation
**4411**	Short/branched chain specific acyl-CoA dehydrogenase, mitochondrial	P45954	44681/5.83	69	12				Lipid metabolism
**5308**	Serine/threonine-protein phosphatase PP1-alpha catalytic subunit	P62136	38229/5.94	239	29				Protein phosphorylation
**5414**	Galactokinase	P51570	42702/6.04	131	14				Glycometabolism
**6205**	Peroxiredoxin-6	P30041	25133/6.60	70	18				Antioxidant defense
**6211**	Platelet-activating factor acetylhydrolase IB subunit gamma	Q15102	25832/6.33	136	26				Lipid metabolism
**6403**	Septin-2	Q15019	41689/6.15	123	21				Cytoskeleton
**6707**	Succinate dehydrogenase [ubiquinone] flavoprotein subunit, mitochondrial	P31040	73672/7.06	170	25				Respiration
**8012**	Cofilin-1	P23528	18719/8.22	128	31				Signal transduction

^a^ SwissProt accession number.

^b^ A: control group. B: FFA group. C: FFA + EGCG (50 μM) group.

### Validation of differentially expressed proteins by western blotting

**Figure 3:**
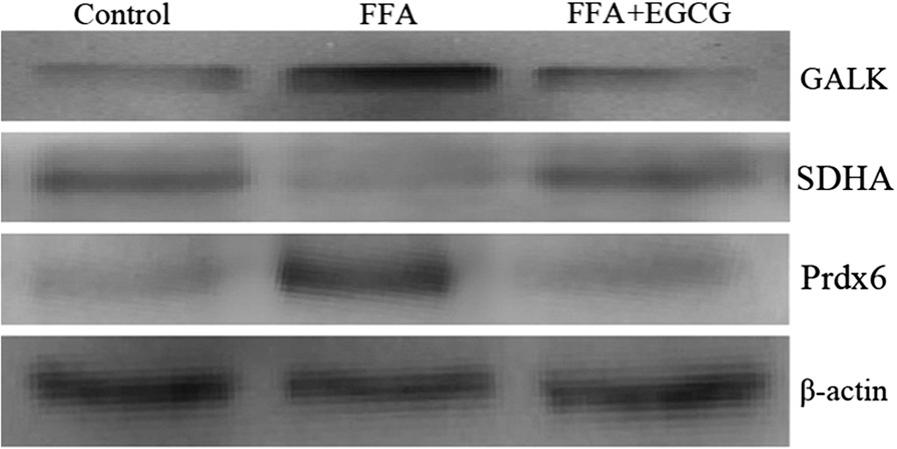
**Validation of Prdx6, GALK, and SDHA expression levels in HepG2 cells by Western blotting.** The expression levels of Prdx6, GALK and SDHA in the control, FFA and FFA + EGCG groups were detected by Western blotting. β-actin was used as a loading control. Similar results were found in the three independent experiments and the representative results are shown.

Western blotting analysis was performed in triplicate to confirm the differentially expression proteins found in the proteomic analysis. FFA-induced HepG2 cells were treated with 50 μM EGCG for 24 h. Equal amounts of total proteins from different treated cells were used for western blotting analysis. The results (Figure 3) suggested that the expressed levels of peroxiredoxin-6 (Prdx6) and galactokinase (GALK) were significantly lower (*p* ≤ 0.05) and succinate dehydrogenase flavoprotein subunit (SDHA) was significantly higher (*p* ≤ 0.05) in the FFA + EGCG group compared with the FFA group. Therefore, the western blotting results agree with the proteomic results.

## Discussion

Lipid accumulation is the main cause of NAFLD, which results in fatty tissue degeneration [1,19]. This lipid accumulation in hepatocytes results from an imbalance between lipogenesis and lipolysis metabolism causing eventual lipoperoxidative stress and hepatic injury [20]. Previous studies suggest that the inhibitory action of EGCG on lipid accumulation is mediated via the AMP-activated protein kinase (AMPK) pathway [21-23]. Activation of AMPK can induce ATP generation through glycolysis and β-oxidation, and suppress fatty acid and cholesterol syntheses, and gluconeogenesis [24]. However, the inhibitory effect of EGCG on FFA-induced intracellular lipid accumulation at the proteomic level has not been investigated. In this study, we reported a comprehensive proteome, which deduced the target genes of EGCG as well as its inhibitory effect on lipid accumulation in FFA-induced HepG2 cells. Eighteen differentially expressed proteins were successfully identified. These proteins are involved in lipid metabolism, glycometabolism, antioxidant defense, respiration, cytoskeleton organization, signal transduction, DNA repair, mRNA processing, and iron storage, or are chaperone proteins. This indicates that these physiological processes may play a role in the mechanism by which EGCG inhibits cellular lipid accumulation in FFA-induced HepG2 cells.

### Proteins involved in redox regulation and energy metabolism

After treatment with EGCG, two differentially expressed proteins related to redox and respiratory regulation were identified in HepG2 cells. Prdx6 is the sixth mammalian member of the peroxiredoxin family, which has an important role in antioxidant defense [25,26]. Several studies reported that an increase in reactive oxygen species (ROS) could lead to AMPK activation [27,28]. EGCG significantly induced generation of ROS and reduced glutathione (GSH) in cells [21-23]. AMPK is thought to be a novel target for the treatment of obesity and type II diabetes [29]. Activation of AMPK in liver and skeletal muscle leads to the stimulation of fatty acid oxidation and inhibition of lipogenesis, glucose production and protein synthesis [30,31]. However, these effects could be eliminated by ROS scavenger, N-acetylcysteine, treatment [22,23,28]. In our proteomic experiment, Prdx6 was down-regulated by EGCG treatment. This may be a reason for the increasing ROS levels that lead to AMPK activation, preventing lipid accumulation in cells. Succinate dehydrogenase (SDH or ubiquinone) is an enzyme that controls the transcription of metabolism-related genes in mitochondria and promotes metabolism of glucose and lipids [32,33]. SDH is related to oxidative metabolism and is an indicator of oxidative capacity in cells [34]. Prior studies have found that a reduction in oxidative enzymes is correlated with a reduced capacity for lipid oxidation and increased risk for obesity [35,36]. The mRNA expression levels of SDH are reduced in mice with type II diabetes mellitus and obesity [37]. Several studies have also shown that weight loss and exercise could result in a significant increase in SDH activity [38,39]. Our proteomic analysis showed that SDHA was up-regulated by EGCG treatment in FFA-induced HepG2 cells, indicating that EGCG increased lipid oxidation by enhancing energy expenditure and fat oxidation in the mitochondria of HepG2 cells.

### Proteins involved in lipid metabolism

Proteins involved in lipid metabolism were changed significantly by EGCG treatment in HepG2 cells. Platelet-activating factor acetylhydrolase (PAFAH), a unique member of the phospholipase A2 superfamily, is characterized by its ability to specifically hydrolyze platelet-activating factor (PAF) and glycerophospholipids [40]. PAF is a potent pro-inflammatory phospholipid that activates cells involved in inflammation and stimulates ROS generation [41-43]. Down-regulation of platelet-activating factor acetylhydrolase IB subunit gamma (PAFAH1B3) by EGCG treatment might lead to an increase of PAF, which could stimulate the generation of ROS and activating AMPK in HepG2 cells. Short/branched-chain acyl-CoA dehydrogenase (SBCAD) catalyzes the first step in the mitochondrial β-oxidation of L-2-methylated short acyl-CoA compounds [44]. SBCAD deficiency is characterized by accumulation of 2-methylbutyrylglycine (2MBG), which induced an increase of lipid oxidation and a decrease of antioxidant defenses (decreased GSH) in rat [45]. In our proteomic experiment, SBCAD was down-regulated in the EGCG-treated group. This might result in a decrease of antioxidant defense and a high level of cellular ROS, which could activate AMPK to prevent cellular lipid accumulation.

### Proteins involved in glycometabolism

Two proteins involved in glycometabolism were down-regulated by EGCG treatment. GALK catalyzes the phosphorylation of α-galactose to galactose-1-phosphate in the second step of the Leloir pathway, a metabolic pathway found in most organisms for the catabolism of β-galactose to glucose-1-phosphate [46]. Glucose-1-phosphate could be metabolized to glucose-6-phosphate providing substrate for the pentose phosphate pathway, which generates ribose-5-phosphate and NADPH for the biosynthesis of fatty acids and sterols [47]. A study reported that unsaturated fatty acids could increase the activation of galactokinase and galactose-1-phosphate uridyltransferase, and stimulate sterol biosynthesis from galactose [48]. Down-regulation of galactokinase reduces ribose-5-phosphate and NADPH from the pentose phosphate pathway, resulting in a reduction of the biosynthesis of fatty acids and sterols in FFA-induced HepG2 cells. Lysosomal protective protein (PPCA) appears to be essential for the activity of β-galactosidase, which catalyzes the hydrolysis of β-galactosides into β-galactose and glucoside. PPCA associates with β-galactosidase and exerts a protective function necessary for its stability and activity [49]. Down-regulation of PPCA could reduce the activity of β-galactosides and decrease β-galactose for the Leloir pathway, inhibiting the biosynthesis of fatty acids and sterols from galactose [47,48].

### Proteins involved in biological regulation and signal transduction

Some proteins involved in biological regulation and signal transduction were also identified in HepG2 cells, including serine/threonine protein phosphatase-alpha catalytic subunit (PP-1A), phosphoserine phosphatase (PSP), 14-3-3 protein gamma and prohibitin (PHB). PP-1A and PSP belong to the phosphoprotein phosphatase family, which remove the phosphate from the serine or threonine residues of phosphoproteins [50]. Previous study reported that a glucose-induced activation of the transcription of the FAS gene was markedly reduced by okadaic acid, an inhibitor of protein serine/threonine phosphatases 1 (PP1) and PP2A, and by AICAR, a cell-permeable activator of the AMPK [51]. These results indicated that the reduction of the FAS gene involves a phosphorylation/dephosphorylation mechanism and AMPK activation. Therefore, down-regulation of PP-1A and PSP might indicate an inhibition of lipid biosynthesis and an activation of AMPK in HepG2 cells by EGCG treatment. 14-3-3 protein gamma belongs to the 14-3-3 protein family, which has the ability to bind many functionally diverse signaling proteins, including kinases, phosphatases, and transmembrane receptors [52,53]. The transcriptional co-activator transducer of regulated CREB activity 2 (TORC2) is a pivotal component of gluconeogenesis [54]. Phosphorylated TORC2 is sequestered in the cytoplasm via a phosphorylation-dependent interaction with 14-3-3 proteins and degraded by 26S proteasome to inhibit the gluconeogenic program [55]. A report suggested that inhibition of enhanced gluconeogenesis induced by high-fat and high-fructose diet could improve lipid metabolism and hepatic steatosis in mice [56]. Thus, up-regulation of 14-3-3 protein by EGCG treatment in FFA-induced HepG2 cells might indicate that the hepatic steatosis induced by overloaded FFA was improved through inhibition of gluconeogenesis. PHB comprise two evolutionarily conserved proteins, prohibitin-1 (PHB1) and prohibitin-2 (PHB2), and are present in a high molecular-weight complex in the inner membrane of mitochondria [57,58]. PHB1 decreased insulin-stimulated oxidation of glucose and fatty acids, implying that PHB1 may play a role in promoting fat accumulation [59]. A microarray analysis showed that the expression levels of PHB were increased during 3T3-L1 cell adipogenesis [60]. Several studies showed that depletion of PHB1 or PHB2 in C. *elegans* or 3T3-L1 cells could significantly decrease adipose accumulation and adipogenesis [61]. In the current study, EGCG decreased the expression of PHB, which might be one of the reasons that EGCG could inhibit lipid accumulation in FFA-induced HepG2 cells.

## Conclusions

**Figure 4:**
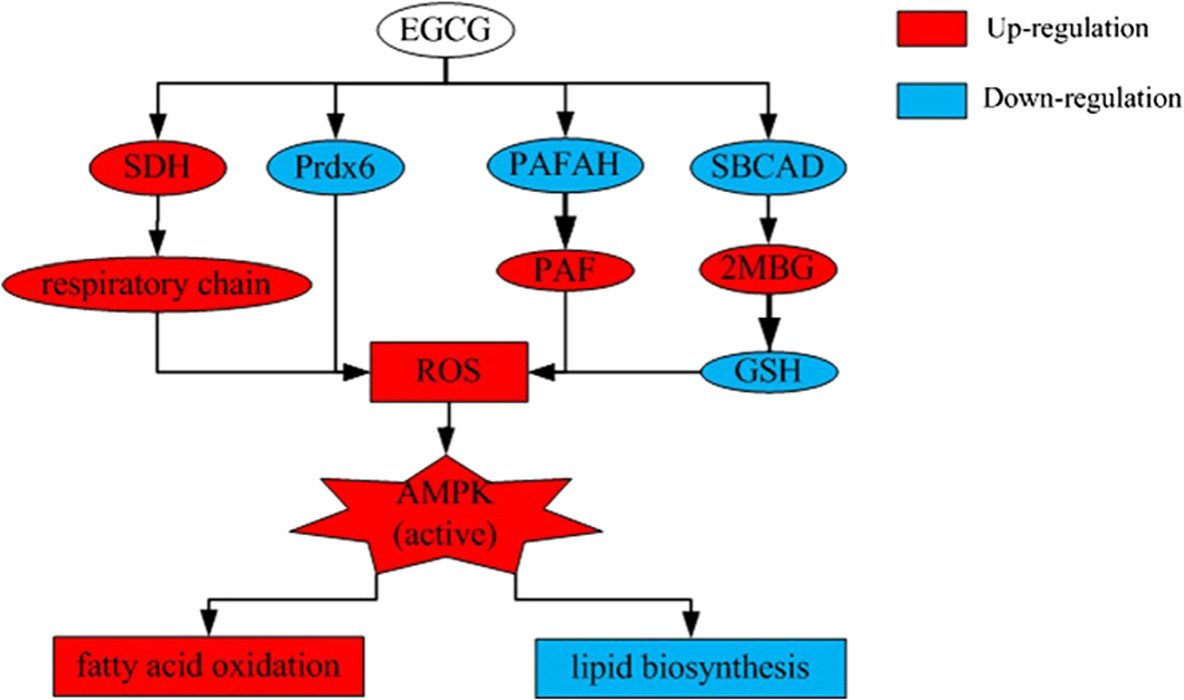
**A simple model of the proposed mechanism by which EGCG inhibits lipid accumulation in FFA-induced HepG2 cells.** Arrowheads indicate the direct or indirect interactions. The up-regulated proteins are marked with red and the down-regulated proteins are marked with blue.

In summary, this study demonstrates that EGCG can significantly suppress the lipid accumulation in FFA-induced HepG2 cells. Using a proteomic approach, we identified 18 differentially expressed proteins responsive to EGCG treatment involving multiple cellular processes. From the proteomic analysis, we supposed that EGCG reduced cellular lipid accumulation in FFA-induced HepG2 cells through the activation of AMPK resulting from the generation of ROS. The induction of ROS may be because EGCG regulated the antioxidant defense system in HepG2 cells (Figure 4). Activation of AMPK shifted some FFA toward oxidation, away from lipid and triglyceride storage, and suppressed hepatic gluconeogenesis in HepG2 cells. Findings of this study provide information to improve our understanding of the molecular mechanisms of the inhibition of lipid accumulation by EGCG in HepG2 cells.

## Methods

### Materials

All chemicals used were of analytic grade. EGCG was obtained from Sigma (St. Louis, MO, USA), the purity of EGCG was ≥95%. Oleic acid and palmitic acid were also obtained from Sigma. Antibodies to β-actin and Prdx6 were obtained from GeneTex (Irvine, CA, USA). Antibodies to SDHA and GALK were obtained from Abcam (Cambridge, UK).

### Cell culture and EGCG stimulation

Experiments were approved by the ethics committee of Tsinghua University. HepG2 cells (human liver hepatocellular carcinoma cell line) were obtained from the cell bank of the Type Culture Collection of Chinese Academy of Sciences, and were cultured in DMEM medium (Thermo, South Logan, UT, USA) with 10% fetal calf serum, 100 U/mL penicillin, 100 μg/mL streptomycin, and 2 mM L-glutamine and kept at 37°C in a humidified atmosphere (Nuaire, Plymouth, MN, USA) with 5% CO_2_. To induce fat-overloading, HepG2 cells were exposed to 1 mM of FFA mixture (OA / PA, 2:1) mixed with 1% FFA-free bovine serum albumin [17,28,62]. Stock solutions of 50 mM FFA were prepared as reported previously [63]. When the cells reached 70% confluence, they were incubated in serum-free medium for 24 h before treatments. Then, the cells were stimulated with 1 mM FFA and co-treated with EGCG for 24 h.

### Cell proliferation and apoptosis assay

The effect of EGCG on cell proliferation was assessed using MTT assay as previously reported [64]. Briefly, HepG2 cells were plated in 96-well plates (5 × 10^3^ cells/well) and treated with 1 mM FFA with various concentrations of EGCG for 24 h. Then cells were incubated with 100 μL MTT solution (0.5 mg/mL MTT in PBS buffer) for 4 h. The absorbance was measured with a Multiskan MK3 (Thermo) at 490 nm. Apoptotic cells were detected using flow cytometry with PI/annexin V staining [64]. Cells were seeded in six-well plates (4 × 10^6^ cells/well) and co-incubated with 0, 50, and 100 μM of EGCG and 1 mM FFA for 24 h. After harvesting with 0.25% trypsin, cells were resuspended with 200 μL binding buffer and incubated with 10 μL Annexin V-FITC and 5 μL PI for 15 min at room temperature. Sample solutions were adjusted to 500 μL using binding buffer and analyzed with a FACScan flow cytometer (Becton Dickinson Biosciences, San Jose, CA, USA) within an hour of sample preparation. The data were analyzed by Modfit3.0 (Verity Software House, Topsham, ME, USA). All treatments were performed in triplicate.

### Oil Red O staining

The Oil Red O (ORO) staining was according to Amacher et al. [65,66]. Briefly, the cells were washed twice with ice-cold PBS and fixed with 10% formalin for 1 h, and stained with Oil Red O solution for 2 h at room temperature. After staining, cells were washed twice with distilled water to remove unbound dye. To quantitate the intracellular lipid content levels, isopropanol was added to each sample shaken at room temperature for 5 min, and samples were read spectrophotometrically at 520 nm.

### Triglycerides and cholesterol assay

For lipidic determinations, homogenates from cells were extracted according to a Heider et al. [67]. Briefly, each sample was homogenized with isopropyl alcohol. The resulting mixture was shaken at room temperature for 1 h and then centrifuged at 1200 × g for 10 min. The supernatant was collected for analysis of hepatic TG and TC content. The residue was dissolved in 0.1 M sodium hydroxide and an aliquot was taken for protein determination with an RC DC Protein Assay Kit (Bio-Rad, Hercules, CA, USA). Triacylglycerol and cholesterol were measured using enzymatic method kits (Cell Biolabs, Inc. San Diego, CA, USA) according to the manufacturer’s instructions.

### Protein extract and sample preparation for 2-DE

The HepG2 cells were harvested by trypsinization and washed twice with ice-cold PBS buffer. The cells were lysed in lysis buffer (7 M urea, 2 M thiourea, 4% [w/v] CHAPS, 65 mM DTT, 2% [v/v] Bio-Lyte pH [3-10], 2% protease inhibitor cocktail), mixed by vortexing, kept in an ice bath for 2 h, then sonicated in an ice bath. The sample was clarified by centrifugation at 15000 × g for 1 h at 4°C, and the supernatants stored at −80°C until use for 2-DE. Protein content was quantified using the RC DC Protein Assay Kit (Bio-Rad).

### 2-DE and image analysis

About 1.3 mg protein dissolved in 350 μL rehydration buffer was applied to IPG strips (17 cm, pH 3–10, Bio-Rad), which was allowed to rehydrate for 13 h at 50 V (20°C). Subsequently, isoelectric focusing (IEF) was performed at using a Protean IEF Cell (Bio-Rad) under the following conditions: 250 V for 1 h with a slow increase in voltage, 500 V for 1 h with a slow increase in voltage, 1000 V for 1 h with a slow increase in voltage, 10000 V for 5 h with a linear increase in voltage, and maintained at 10000 V until 60000 Volt-hours (Vh) was reached. After IEF, the strips were equilibrated for 15 min in equilibration buffer I (0.375 M Tris–HCl pH 8.8, 6 M urea, 2% SDS, 20% glycerol, 1% DTT), then re-equilibrated in buffer II containing 2.5% iodoacetamide instead of DTT for 15 min. The strips were transferred onto 12% polyacrylamide gels for SDS-PAGE. Electrophoresis was performed using the PROTEAN II xi Cell system (Bio-Rad) at 10 mA per gel for 30 min, followed by 30 mA until the bromophenol blue marker reached the end of the gel. Gels were run in triplicate for each sample. The gels were stained with modified colloidal CBB G-250 [68] and were scanned using Quantity One 4.6.9 (Bio-Rad) with a Bio-Rad GS800 scanner. Image and statistical analysis was performed with PDQuest 8.0.1 (Bio-Rad) as previously reported [69]. In the quantitative analysis, 1.5 and 0.5 were chosen as the upper and lower limits, respectively. Student’s *t*-test and a significance level of 95% were used for the statistical analysis of the gels. Each sample was performed in triplicate gels.

### Protein in-gel digestion and identification

Protein spots were manually excised from the gel, and digested as previously reported [69]. Mass spectrometric analysis was performed with an Ultraflex MALDI TOF/TOF mass spectrometer (Bruker Daltonik, Bremen, Germany), under the control of FlexControl™ 2.2 software (Bruker Daltonik GmbH). The TOF spectra were recorded in the positive ion reflector mode in a mass range from 800–4000 Da. Ten subspectra with 30 shots per subspectrum were accumulated to generate one main TOF spectrum. After automated assessment of the search results with MASCOT software (Matrix Science, London, UK), the samples not unambiguously identified by PMF were automatically submitted to MS/MS analysis using the LIFT technology in TOF/TOF. A maximum of three strongest peaks of the TOF MS spectrum per sample were chosen for MS/MS analysis. Three subspectra with 50 shots per subspectrum for precursor ions and 15 subspectra with 50 shots per subspectrum for fragment ions were accumulated to produce one main MS/MS spectrum. After the automated analysis, remaining unidentified samples were manually analyzed.

### Database searching

Protein identification from MS/MS sequencing spectra was accomplished using the MASCOT database search engine (Matrix Science, London, UK). The searching parameters were set as follows: taxonomy, Homo sapiens; database, NCBInr/Swiss-Prot; enzyme, trypsin; fixed modifications, carbamidomethyl (C); variable modifications, oxidation (M); no restrictions on protein mass; allow up to one missed cleavages. Mass values, Monoisotopic; Peptide Mass Tolerance was set as ±50 ppm; Fragment Mass Tolerance was set as ±0.5 Da. Positive protein identification was based on standard MASCOT criteria for statistical analysis of the LC-MS/MS data. The peptide assignments in the database search results were manually inspected for validation.

### Western blotting analysis

The HepG2 cells were harvested by trypsinization and washed twice with ice-cold PBS buffer. The cells were lysed in RIPA buffer (25 mM Tris–HCl pH 7.6, 150 mM NaCl, 1% NP-40, 1% sodium deoxycholate, 0.1% SDS, 2% protease inhibitor cocktail). Equal amounts of protein were separated on a 12% SDS-polyacrylamide gel, blotted onto a PVDF membrane, which was blocked for 1 h at 25°C with 5% wt/vol BSA/TBST (10 mM Tris HCl, pH 7.4, 140 mM NaCl, 0.1% Tween-20) and then incubated with the primary antibody for Prdx6 (1:3000), SDHA (1:1000) and GALK (1:1000) at 4°C overnight. After washing with TBST, the membranes were incubated with the appropriate secondary antibodies for 1 h at 37°C and detected by immuno-staining. After the membranes were scanned, the signal intensity of each band was determined using FluorChem FC2 (Alpha Innotech Co., Ltd, San Leandro, CA, USA).

### Statistical analysis

All results were expressed as mean ± SD and analyzed by student’s *t*-test. A *p* ≤ 0.05 was considered to be statistically significant.

## Abbreviations

EGCG: (−)-epigallocatechin-3-gallate; 2-DE: Two-dimensional gel electrophoresis; MALDI-TOF/TOF MS: Matrix-assisted laser desorption ionization time-of-flight mass spectrometry; FFA: Free fatty acid; OA: Oleic acid; PA: Palmitic acid; TG: Triglycerides; TC: Cholesterol; AMPK: AMP-activated protein kinase; Prdx6: Peroxiredoxin-6; ROS: Reactive oxygen species; SDH: Succinate dehydrogenase; SDHA: Succinate dehydrogenase flavoprotein subunit; PAFAH: Platelet-activating factor acetylhydrolase; PAF: Platelet-activating factor; PAFAH1B3: Platelet-activating factor acetylhydrolase IB subunit gamma; SBCAD: Short/branched-chain acyl-CoA dehydrogenases; 2MBG: 2-methylbutyrylglycine; GSH: Glutathione; GALK: Galactokinase; PPCA: Lysosomal protective protein; PP-1A: Serine/threonine protein phosphatase-alpha catalytic subunit; PSP: Phosphoserine phosphatase; FAS: Fatty acid synthase; TORC2: Transducer of regulated CREB activity 2; PHB: Prohibitin.

## Competing interests

The authors declare that they have no competing interest.

## Authors’ contributions

ZHL conceived of the study, and participated in all the experiment. QL participated in the 2-DE experiments, mass spectrometry analysis and western blotting analysis. JAH participated in participated in the design of the study. QLL participated in the cell experiments. YJY participated in the 2-DE experiments. HYL participated in western blotting analysis. WJX participated in the critical review of the manuscript. YL participated in the protein identification. SZ participated in the protein identification. BT participated in cell experiments. GAL conceived of the study, and participated in its design and coordination. All authors read and approved the final manuscript.
